# Patient activation in adults with visual impairment: a study of related factors

**DOI:** 10.1186/s12889-024-18856-5

**Published:** 2024-06-14

**Authors:** Esma Özkan, Özgü İnal Özün, Ayşe Göktaş, Bayazıt İlhan

**Affiliations:** 1 Department of Occupational Therapy, Gülhane Faculty of Health Sciences,, University of Health Sciences Turkey, Keçiören, Ankara, 06018 Türkiye; 2 Neurological Physiotherapy-Rehabilitation, Gülhane Faculty of Physiotherapy and Rehabilitation, University of Health Sciences Turkey, Ankara, Türkiye; 3 University of Health Sciences Turkey Ulucanlar Eye Training and Research Hospital, Ankara, Türkiye

**Keywords:** Patient activation, Visual impairment, Self-efficacy, Self-management, Healthy life awareness, Social relations, Environment

## Abstract

This study aims to analyze variables related to patient activation in 78 individuals with visual impairment. The Patient Activation Measure (PAM) scores of participants showed no differences between males and females. It was found that the individuals living in urban areas, and participants with higher income and education levels had higher PAM scores. Still, the difference between the groups was statistically insignificant (*p* > 0.05). The PAM scores of the visually impaired individuals reflect taking action level of activation (66.51 ± 18.14-PAM level 3). There was a moderately significant relationship between PAM scores and visually impaired individuals’ self-management, self-efficacy, healthy life awareness, social relations, and environment (*p* < 0.001). We found that the variables included in the regression model (marital status, self-management, self-efficacy, healthy life awareness, social relations, and environment) explained 72.2% of the PAM score. Individuals with visual impairment can be given training on self-management, self-efficacy, healthy life awareness, and quality of life associated with social relations and environment to develop positive health behaviors.

## Introduction

Visual impairment is a chronic condition whose prevalence increases with age [1], and it is considered a public health problem that impacts communities both socially and financially. According to the World Health Organization (WHO) reports, the global prevalence of visual impairment has been reported as approximately 285 million people. Of these, approximately 39 million people experience total loss of sight, and 246 million have low vision [2]. Visual impairment, an important cause of disability, increases the risk of death; it negatively affects all functions and self-care activities, autonomy, health-related self-management, self-control and self-efficacy; and it decreases health-related quality of life [3–9].

Patient activation is defined as ‘an individual’s tendency to engage in adaptive health behaviours that lead to better health outcomes’. A recent study shows that higher patient activation is associated with the majority of better clinical indicators, healthier behaviours, preventive care and lower future costs [10]. If the patient’s activity level is known, the level is started with an appropriate target and increased step by step, so the patients will be able to experience small successes and build the confidence and skills necessary for effective self-management [11].

The greatest success in the effective management of chronic diseases can be achieved with an active patient who participates in their self-care [12–14]. Patient activation is having the knowledge, skill and confidence to self-manage disease symptoms and health problems, participate in activities that maintain or improve functioning, and be an active participant in one’s own health care [11, 13]. Therefore, patients’ involvement in decision-making about their own health plays a leading role in successful self-management and health promotion [12].

The effect of vision loss on activation varies based on many factors. Age of onset of vision loss, duration of life with vision loss, and patient perception of the impact of visual impairment—rather than clinical measures—may be more important factors in understanding the importance of vision loss on activation [15, 16]. Cognition in accepting the disease requires acceptance of low vision and confidence in living with limitations and adapting to them. It is suggested that the adaptive and maladaptive cognitions demonstrated in chronic disease states may be important for fully understanding individual differences in adaptation to chronic diseases. Acceptance refers to accepting the need to adapt to a chronic illness while perceiving the ability to tolerate the unpredictable, uncontrollable nature of the illness and cope with its negative consequences [17]. A body of evidence demonstrates that greater acceptance of visual impairment is associated with improved psychological adaptation [18–20]. Moreover, the patient’s interpretation, perception and evaluation of their illness as an individual, as well as their emotional and behavioural reactions, are the factors that determine the patient’s way of coping, the development of psychosocial difficulties and psychiatric disorders, and the quality of life [21]. Owsley et al. have shown that, even though most of the comments of individuals with age-related macular degeneration (AMD) were negative, the number of these negative comments was not related to disease severity [22]. This data indicates that how individuals with vision loss react to the medical condition is more significant than the rate of vision loss. Vision loss is characterized by its progressive nature and negative impact on daily life. As a result, patients may encounter persistent psychological stress stemming from anxiety or fear, in addition to secondary repercussions including social isolation and depression [23]. Prolonged mental stress can exacerbate vision loss, even though it is an obvious consequence of the condition. Regarding this, it is possible to assert that tension is both the catalyst and the consequence of visual impairment. This psychosomatic perspective holds significant value in advocating for clinical practices that involve coping mechanisms, self-management, awareness, and access to social and environmental support for those who are experiencing vision loss [24, 25].

Prevention of complications and favourable prognosis are possible with effective management of chronic diseases. According to the active patient concept as defined by Hibbard et al., the individual believes that they have an important role in self-management, cooperates with supportive people, maintains their health, and knows how to manage their condition, protect their functions, and prevent regression in health status [11]. In addition, the individual has the ability to behaviourally maintain their current state, cooperate with the health team, maintain and protect health functions, and access high-quality care appropriately [12, 13]. When patients are actively engaged in their self-care, care experiences and outcomes improve [26–28].

Evidence on patient activity is critically important, especially in individuals with chronic diseases, because individuals need to follow complex treatment regimens, monitor their condition, make lifestyle changes, and be decision-makers in their care. Such evidence is a prerequisite for the extensive adoption and implementation of strategies to support greater activation in patients. Although many studies have investigated patient activation in many different chronic diseases [29–31], the ability of individuals with vision loss to participate actively in the management of their health care has not been extensively studied, and the evidence on this subject is insufficient [16, 32]. Based on the absence of studies on this subject, we aimed to study and examine the variables related to patient activation in adults with visual impairment. We suggest that the results of the study will guide the diagnosis and rehabilitation process of the disease in individuals with visual impairment.

## Methods

The study was performed according to the guidelines of the Declaration of Helsinki. The study was approved by the University of Health Sciences Gülhane Scientific Research Ethics Committee (2021 − 400/25.11.2021). Individuals who applied to the Ulucanlar Eye Training and Research Hospital Ophthalmology Unit and met the inclusion criteria were referred to study. The interviews were held between January and April 2022. All participants who met the inclusion criteria and gave informed consent completed the face-to-face interview. The scale items were read aloud, and the participants were asked to choose the option that they found most suitable. The answers given by the participants were recorded by the researchers.

### Participants

The study group consisted of individuals over the age of 18. Having a visual acuity worse than 20/40 and having a diagnosis causing visual impairment were determined as inclusion criteria. Individuals with any psychological disorders and communication problems were excluded from the study.

### Instruments

Socio-demographic information form, Patient Activation Measure (PAM), Self-Control and Self-Management Scale (SCMS), General Self-Efficacy Scale (GSES), Healthy Life Awareness Scale (HLAS) and World Health Organization Quality of Life Assessment (WHOQOL-BREF) were applied to the individuals participating in the study.

The sociodemographic form included questions such as the participants’ age, gender, marital status, education level, place of residence, income level, and age at the onset of vision loss. In addition, the participants’ vision loss rates and general disability rates included in the physician committee report issued by the Ministry of Health were also recorded.

#### Patient activation measure (PAM)

PAM was developed by Hibbard et al. in 2004 [13] in patients with chronic disease in order to detect and evaluate patient activity level, and in 2005 [11], Hibbard et al. studied a short version of the scale in patient group with a chronic disease. PAM is a valid, highly reliable, one-dimensional, Guttman-type scale. The scale has 22 items, but in this study, we used the Turkish version, which consists of 13 items. The scale scoring system is as follows: *Strongly Agree* = 4 points, *Agree* = 3 points, *Disagree* = 2 points, *Strongly Disagree* = 1 point, and *I Don’t Know/Can’t Evaluate* = 0 points. The activity scores obtained from the measurement tool range from 0 to 100 points. So, the results were interpreted as: Stage 1 = lowest activity (belief in the importance of taking an active role): < 47; Stage 2 (knowledge and confidence to take action) = 47–55; Stage 3 (taking action) = 55–72; Stage 4 = highest activity (keeping routine even under stress): > 72.5. The Cronbach’s alpha internal consistency coefficient of the original scale is 0.91, while this number is 0.81 in the Turkish version [13, 33].

#### Self-control and self-management scale (SCMS)

The Self-Control and Self-Management Scale was developed by Mezo in 2008 [34], and the Turkish testing of the scale’s validity and reliability was performed by Ercoşkun in 2016 [35]. SCMS is an adult self-assessment tool that was developed to measure the general characteristics of self-control and self-management skills. It has a cognitive and behavioural structure and was successfully applied and evaluated during the scale development stage [34, 35]. SCMS is a process-oriented scale that independently evaluates each of the three components of the self-management structure [36, 37]. It consists of three sub-dimensions: Self-Reinforcing (SR), Self-Evaluating (SE) and Self-Monitoring (SM). The total score ranges from 0 to 80. A high score indicates high self-management and self-control [35].

#### General self-efficacy scale (GSES)

The General Self-Efficacy Scale-Turkish form is a valid and reliable tool that measures the general self-efficacy of people 18 and older who are at least primary school graduates. In the study, the Likert-type scale (consisting of five different responses ranging from ‘Not at All’ to ‘Exactly True’) was used for the question ‘How well does this describe you?’ The score of each question is between 1 and 5. Items 2, 4, 5, 6, 7, 10, 11, 12, 14, 16 and 17 in the scale are reverse scored. The total score of the scale ranges between 17 and 85. A higher score indicates more self-efficacy [38].

#### The healthy life awareness scale (HLAS)

The HLAS is a 15-item and five-point, Likert-type scale. The lowest score is 15, and the highest score is 75. A high score on the scale is considered a high level of healthy living awareness. Cronbach’s alpha value was 0.813, test-retest reliability coefficient was determined to be 0.849, and the scale was proven to be highly reliable [39].

### World Health Organization Quality of Life Assessment (WHOQOL-BREF)

The health-related quality of life scale is a scale developed by WHO [40] that measures a person’s well-being and allows cross-cultural comparisons. Eser et al. [41] were tested Turkish validity and reliability. The scale measures physical, spiritual, social and environmental well-being and consists of 26 questions. Each area expresses the quality of life in its own area, independently of each other. As the score increases, the quality of life increases. Cronbach’s alpha internal consistency coefficients of the scale were obtained as 0.76 in the physical health dimension, 0.67 in the psychological health dimension, 0.56 in the social relations dimension and 0.74 in the environmental dimension. Test-retest reliability varies between 0.51 and 0.81 [42]. Social relations and environment sub-dimensions were used in our research.

### Sample size

At the beginning of the study, the number of participants was determined using the G*Power (version 3.1.9.4) package programme. The sample size was calculated according to the multivariate linear regression analysis with six variables that predicted the PAM score, which was the primary variable. When the Cohen f^2^ effect size index was 0.21, the type 1 error rate was 0.05, the power was 0.80, and the sample size was determined as a minimum of 76 individuals with vision loss.

### Statistical methods

For descriptive statistics, mean ± standard was used for continuous data, and frequency and percentage were used for categorical data. Conformity of continuous data to normal distribution was checked by the Kolmogorov-Smirnov test and graphical analysis (box-line plot, Q-Q Plot). The difference between the two groups was evaluated using the t-test in independent groups with normal distribution, and with the Mann-Whitney U test for those without normal distribution. The distribution of scale scores in groups of three or more was evaluated with one-way ANOVA for those with normal distribution, and with Kruskal-Wallis test for those without normal distribution.

Multiple linear regression analysis was performed with the Enter method to obtain the estimation model. From the linear regression assumptions, conformity to normal distribution was examined by the Kolmogorov-Smirnov test, and linear relationship was examined by scatter plot. The adequacy of the model was evaluated by multicollinearity Variance Inflation Factor (VIF) analysis. Autocorrelation between errors was analysed by Durbin-Watson (D-W) test, effective observations were analysed by Covariance Ratio, and distant observations were analysed by Cook’s distance. Variance homoscedasticity, normal distribution of errors, and extremely distant and outlier observations were examined with residual plots [43]. In case of multicollinearity among the independent variables included in the model, Ridge Regression (RR), one of the biased regression techniques, was used instead of ordinary least squares (OLS) regression.

Ridge regression analysis was performed using the NCSS (version 21.0.3) package program. IBM SPSS 21 (IBM SPSS Inc, Chicago, IL) program was used for all other analyses. The statistical significance level was taken as 0.05 [44].

## Results

The study was completed with 78 patients. The mean age of the individuals was 44.87 ± 16.33 years, 49 (62.8%) were male, and 29 (37.2%) were female. Participants were diagnosed with retinal/macular dystrophy (*n* = 25), optic neuropathy/atrophy (*n* = 18), glaucoma (*n* = 14), nystagmus (*n* = 8), retinopathy of prematurity (*n* = 4) and others (*n* = 9).

There was no statistically significant difference between the mean scores of PAM scale of female and male participants (U = 641.50, *p* = 0.475). The distribution of PAM scores based on the education levels of the participants was evaluated using the Kruskal-Wallis test. The mean score of those with undergraduate and graduate education (68.82 ± 16.90) was higher than those with primary and high school education (61.42 ± 21.76 and 66.83 ± 16.10, respectively). However, the difference was not significant (χ2 = 2.045, *p* = 0.360). The distribution of scale scores to income levels was evaluated using the Kruskal-Wallis test. The mean score (71.49 ± 14.95) of those with an income above and twice the minimum wage (*n* = 26) was higher than other income groups, but the difference was not statistically significant (χ2 = 3.989, *p* = 0.136). Mean PAM scores of individuals living in the urban and smaller residential areas were evaluated using the student’s t test. The mean score of those living in urban areas (67.81 ± 17.63) was higher than those living in smaller residential areas (61.06 ± 19.82), but the difference was not statistically significant (t = 1.302, *p* = 0.197) (Table 1).

**Table 1:** Descriptive statistics of PAM total scores, socio-demographic and other characteristics

Characteristics		Mean ± SD (Min-Max)	Statistics
Sex	Female (*n* = 29)	67.21 ± 19.69 (20.50–100)	U = 641.50* *p* = 0.475
	Male (*n* = 49)	66.10 ± 17.35 (22.50–100)	
Educational background	Primary school (*n* = 20)	61.42 ± 21.76 (20.50–100)	χ^2^ = 2.045** *p* = 0.360
	High school (*n* = 16)	66.83 ± 16.10 (27.60–90.70)	
	Undergraduate and above (*n* = 42)	68.82 ± 16.90 (22.60–100)	
Income status	0-2800 TRY^§^ (*n* = 25)	59.36 ± 20.20 (20.50–90.70)	χ^2^ = 3.989 *p* = 0.136
	2801–5600 TRY (*n* = 26)	71.49 ± 14.95 (48.90–100)	
	5601 TRY and above (*n* = 27)	68.34 ± 17.47 (27.60–100)	
Place of residence	Urban (*n* = 63)	67.81 ± 17.63 (20.50–100)	t = 1.302*** *p* = 0.197
	Smaller residential area (*n* = 15)	61.06 ± 19.82 (34.20–100)	
Marital status	Married (*n* = 40) Single (*n* = 38)	79.09 ± 11.71 (60.60–100) 53.27 ± 13.74 (20.50–75)	U = 90.50* *p* < 0.001

* Mann-Whitney U test, ** Kruskal Wallis test, *** Student t test

^§^TRY: Turkish liras (2800 TRY = minimum wage at the time the research was conducted)

When we examined the distribution of PAM levels and mean PAM scores, we showed that the scores of participants were 36.93 ± 9.26 for PAM level 1 (*n* = 12), 52.11 ± 1.80 for PAM level 2 (*n* = 6), 63.81 ± 5.04 for PAM level 3 (*n* = 29) and 83.28 ± 9.99 for PAM level 4 (*n* = 31).

There was a moderate and statistically significant positive correlation between PAM score (66.51 ± 18.14) and SCMS_SM (24.29 ± 5.96), total SCSM (61.75 ± 12.73), total HLAS (58.72 ± 9.57) and total GSES scores (*p* = 0.000). There was a weak and statistically significant positive correlation between PAM score (66.51 ± 18.14) and SCMS_SR (24.29 ± 5.96) and SCMS_SE (19.00 ± 5.25) scores (*p* < 0.05). A moderate positive (0.560 and 0.643) statistically significant relationship was found between PAM Score (66.51 ± 18.14) and WHOQOL-BREF_Environment (48.84 ± 17.26) and WHOQOL-BREF_Social Relations (54.91 ± 19.53) (*p* < 0.001).

There was a very weak positive correlation between the total PAM score and the rate of vision loss (86.44 ± 17.16), rate of disability (80.52 ± 24.60) and age (44.87 ± 16.33) and a very weak negative correlation (*r* = 0.092, *p* = 0.42; *r* = 0.076, *p* = 0.511; *r* = 0.044, *p* = 0.702) between the total PAM score and the age of vision loss (11.27 ± 18.41) which was statistically (*r*=-0.086, *p* = 0.456) insignificant. As a result of the Point Biserial correlation analysis between PAM score and marital status, a highly (0.716) statistically significant relationship was found (*p* < 0.001). No relationship was found between the participant’s age, vision loss rate, disability rate, and age at onset of vision loss and PAM, so only marital status among sociodemographic variables was included in the regression model.

The linearity of the relationship between PAM and other variables was examined by scatter plot. We showed that the relationship between SCMS_SM, SCMS_SE, SCMS_SR and SCMS_Total and PAM was far from linear. Therefore, we performed square transformation to these variables and these variables were included in the multiple linear regression analysis (multiple linear regression analysis based on the ordinary least squares (OLS) method) as they were [45].

Table 2 shows that, by using residual plots, the errors were normally distributed for the model obtained by ordinary least squares (OLS) regression analysis. We established a model that predicts PAM scores with the Enter method. There was no heteroscedasticity problem and no correlation between errors (autocorrelation) (D-W = 1.79). The outliers were analysed with standardised residuals, effective observations with Covariance Ratio, and distant observations with Cook’s distance values. Two extremely observation (observation 25 and 64) were excluded from the dataset [46]. When Table 2 is examined, VIF and Condition Index (CI) values, which are two important indicators of Multicollinearity, are seen. We found that the VIF values of SCMS_total, SCMS_SM, SCMS_SR and SCMS_SE variables were 385.026, 58.257, 80.378 and 64.709, respectively; these values were greater than 10, indicate multicollinearity problems. In order to determine the presence and degree of multicollinearity, Vinod and Ullah has proposed the condition index (CI) based on the largest and the smallest eigenvalues [47]. If CI is smaller than 10, then there is no multicollinearity issue, if CI is between 10 and 30, it is considered to be a multicollinearity issue, and if CI is greater than 30, then it is considered to be a severe multicollinearity issue [45]. The CI value was 2707.33, indicating a severe multicollinearity.

**Table 2:** Ordinary least squares (OLS) regression analysis for total PAM score

PAM score	β	SE(β)	Beta	t	*p*	VIF	Condition Index (CI)
SCMS_total_square	-,025	,016	-1,910	-1,541	,128	385,026	1
SCMS_SM_square	,061	,040	,734	1,522	,133	58,257	2,87
SCMS_SR_square	,085	,058	,836	1,476	,145	80,378	6,06
SCMS_SE_square	,093	,056	,847	1,667	,100	64,709	7,98
HLAS	,138	,171	,075	,809	,421	2,147	10,55
GSES	,468	,158	,290	2,964	,004	2,402	13,12
WHOQOLBREF_Environment	,225	,083	,214	2,715	,008	1,564	14,55
WHOQOL_Social Relations	,155	,080	,169	1,931	,058	1,924	17,15
Marital Status	-12,942	3,250	-,365	-3,983	,000	2,110	2707,33
F(9,66) = 20,525, *p* < 0.001							
R^2^(Adjusted R^2^) = 0.737 (0.701)							

SCMS: self-control and self-management; SE: self-evaluating, SM: self-monitoring, SR: self-reinforcing, GSES: general self-efficacy scale, HLAS: health life awareness scale; WHOQOLBREF: World Health Organization Quality of Life Assessment

Multicollinearity causes the standard errors of the regression coefficients to be high, t-statistic values to be small, and therefore to reach the wrong conclusion that the contribution of the variables to the regression model is insignificant. It causes the multiple linear regression obtained with the OLS method to produce unreliable results. Multicollinearity among independent variables will result in less reliable statistical inferences. Therefore, we used Ridge Regression (RR) analysis developed by Hoerl and Kennard [48], which is a more effective method. Ridge estimators were calculated to eliminate the multicollinearity problem and obtain estimators with small variance [49].

The stability of the estimations to be made with the RR method depends on the determination of the optimum value for Ƙ. In order to determine the optimum value of the Ridge parameter Ƙ, we used the Ridge Trace method proposed by Hoerl and Kennard [48] and chose the least possible Ƙ value as the optimum Ƙ value in the region where the regression coefficients become stationary. According to the Ridge trace plot and Variance Inflation Factor plot obtained as a result of the Ridge regression analysis, we found that the regression coefficients became more stationary after a very small (Ƙ = 0.02) bias constant. A value of Ƙ = 0.02 was chosen, corresponding to the situation where the VIF values suggested by Marquardt and Snee [50] were between 1 and 10.

Table 3 presents Ridge Regression coefficients and standard errors, least squares coefficients and standard errors, R^2^ and standard errors for the bias constant Ƙ = 0.02. The model established for the Ridge Regression was found to be statistically significant (F(9,66) = 19.031, *p* < 0.001). The established model explains 72.2% of the variation in the PAM score variable.

Based on the results in Table 3, OLS regression model of the factors that can affect the PAM total score for the value of Ƙ = 0.02 was established as:

$$\begin{aligned}{\text{Y}} & = 21.411 + ( - 0.025*{\text{X}}1) + (0.061*{\text{X}}2) \\ & \quad + (0.085*{\text{X}}3) + (0.093*{\text{X}}4) \\ & \quad + (0.138*{\text{X}}5) + (0.468*{\text{X}}6) \\ & \quad + (0.225*{\text{X}}7) + (0.155*{\text{X}}8) \\ & \quad + ( - 12.942*{\text{X}}9) \\ \end{aligned}$$

and RR model was established as:

$$\begin{aligned}{\text{Y}} & = 25.445 + ( - 0.002*{\text{X}}1) + (0.006*{\text{X}}2) \\ & \quad + (0.004*{\text{X}}3) + (0.014*{\text{X}}4) \\ & \quad + (0.130*{\text{X}}5) + (0.442*{\text{X}}6) \\ & \quad + (0.241*{\text{X}}7) + (0.156*{\text{X}}8) \\ & \quad + ( - 12.724*{\text{X}}9) \\ \end{aligned}$$

**Table 3:** Ridge regression parameters, standard errors and VIF values for total PAM score. (Ridge Regression; (Ƙ = 0.02))

PAM score	β	SE(β)	Beta	VIF	*R* ^2^	F
SCMS_total_square (X1)	-,002	,001	-0,137	2,717	0.722	F(9,66) = 19,031, *p* < 0.001
SCMS_SM_square (X2)	0,006	0,008	0,068	2,450		
SCMS_SR_square (X3)	0,004	0,009	0,039	1,800		
SCMS_SE_square (X4)	0,014	0,010	0,126	1,782		
HLAS (X5)	0,130	0,167	0,070	1,937		
GSES (X6)	0,442	0,153	0,274	2,134		
WHOQOLBREF_Environment (X7)	0,241	0,081	0,229	1,424		
WHOQOLBREF_Social Relations (X8)	0,156	0,078	0,170	1,741		
Marital Status (X9)	-12,724	3,166	-0,359	1,896		

SCMS: self-control and self-management; SE: self-evaluating, SM: self-monitoring, SR: self-reinforcing, GSES: general self-efficacy scale, HLAS: health life awareness scale; WHOQOLBREF: World Health Organization Quality of Life Assessment

## Discussion

The results of this study provide information about the factors that may affect patient activation in visually impaired individuals. Our study shows that marital status, self-management, self-efficacy, wellness awareness, quality of life related to social relationships and the environment have a significant effect on patient activation. Individuals with high levels of self-management, self-efficacy, healthy life awareness and positive social relations and supportive environment can avoid useless automatic thoughts and habits. They exhibit conscious behaviours in terms of maintaining and improving health, and they can achieve better results by accessing health services [51].

The PAM scores ​​of participants according to socio-demographic variables did not show any differences among visually impaired men and women. Studies on differences in patient activation among men and women with chronic diseases show conflicting results [16, 52–55]. While some studies show higher levels of patient activation in men [45, 52], some do not show any difference between men and women in terms of patient activation [16, 53, 55]. In our study, we demonstrated that as the education level of the participants increased, PAM scores ​​increased. Literature shows that individuals with chronic diseases and a better education have higher levels of activity [56, 57]. In our study, the PAM scores ​​of the participants with higher income levels were also higher. In addition, individuals living in urban areas had higher PAM scores ​​than those living in smaller residential areas, but there was no statistically significant difference between the groups. Studies have shown that patient activation is only moderately related to socioeconomic status, and education and income account for less than 5–6% of the variation in patient activation [55, 58]. Given the considerable potential for promoting activation and enhancing health outcomes among patients from low socioeconomic status, activation-promoting strategies may prove to be especially efficacious [58]. Patient activation is also affected by complex factors, such as quality of life, well-being and self-efficacy [59, 60]. However, not all of these studies have examined some important factors that could influence the association between socio-demographic variables and patient activation. Therefore, these factors may confound the impact of socio-demographic variables on patient activation.

In our study, the PAM scores of the visually impaired individuals had better activation values (66.51 ± 18.14), compared to the values (58.5 ± 15.0) found in the research conducted by Morse and Seiple [16]. When an individual has basic knowledge of their own condition and treatment for taking action and has some experience and success in changing behaviour, they begin to take action, but there may be a lack of confidence and skills to support the new behaviours [11]. When the action phase is supported by brief interventions, individuals’ current activity levels can change positively. Attempts to make patients ask more questions can make a difference in their information-seeking behaviour [12]. Physical symptoms, environmental stimuli and media are among the factors that influence individuals’ protective health behaviours to take action [51]. Using these factors correctly may contribute to the development of the activity levels of visually impaired individuals.

Age of onset of vision loss, the time lived with vision loss, and the patient’s perception of the impact of vision loss—rather than clinical measures—may be key factors in understanding the importance of vision loss on activation [16]. Although the majority of comments on the impact of vision loss in patients with AMD were unfavorable, these negative remarks were not correlated with the severity of the disease [22]. In addition, Morse & Seiple have shown that there was no relationship between visual acuity and patient activation in visually impaired individuals [16]. In the current study, the rate of vision loss, total disability rate, and the age of onset of vision loss were recorded in the visually impaired individuals, and similar to Morse and Seiple’s study [16], there was no relationship between these variables and PAM scores. Our research studied the correlation between marital status, a social variable, and PAM. The findings revealed a positive association between being married and high PAM levels. Being married can be thought of as social support in chronic condition [61, 62]. From this perspective, it may have turned into a positive life situation in terms of patient activation.

In our study, there was a moderately significant relationship between the self-management, self-efficacy, healthy life awareness, quality of life related to social relationships and the environment of visually impaired individuals and PAM scores. Van do et al. have reported that low patient activation levels were associated with low self-efficacy, poor knowledge on heart failure, and low engagement in heart failure self-management behaviours after being discharged from the hospital [63]. In Social Cognitive Theory, self-management is an important factor in self-efficacy, skills and behaviour change [64]. Self-management is defined as the patient’s knowledge, skills, abilities and willingness to manage one’s own health and care [65]. Increased self-efficacy can help patients gain more control over their health outcomes and alleviate some of their concerns about vision loss [66]. One way to approach self-management is to activate the patient to participate in their own care. Patient activation is one of the important steps in addressing self-management and self-efficacy needs in the best possible way for individuals with chronic conditions [67]. Patient activation affects activities of daily living and self-management, and patient activation awareness, knowledge and skills can help improve health care outcomes [60, 63]. In our study, we found that the variables included in the regression model explained 52.9% of the PAM score. According to our model, activation can be explained by self-management, self-efficacy and health awareness. Previous research has shown that more activation is associated with more knowledge of state [28]. We believe that knowing the level of activation in visually impaired individuals will shape self-management programmes in the management of chronic health conditions, and this can improve health outcomes by ensuring patient-specific planning of the programmes.

A comprehensive understanding of the social context surrounding patient activation in chronic diseases has significant ramifications for the development of interventions targeted at enhancing self-management behavior associated with patient activation, as well as for the overall health and well-being of individuals with chronic diseases. Social support is crucial for preserving optimal bodily and mental health [68]. Overall, research suggests that having strong, supportive social networks can help people become more resilient to stress, guard against the emergence of trauma-related psychopathology, lessen the functional effects of trauma-related disorders like post-traumatic stress disorder (PTSD), and lower their risk of illness and death [19, 69, 70]. Our research found a correlation between patient activation levels of individuals with visual loss and social relationships as a sub-dimension of quality of life. Additionally, social relations were shown to be significant in the developed regression model. According to these findings, we believe that family members and friends can assist in the self-management of vision loss by offering intermittent guidance, offering tangible support that aids in self-management, such as emotional support, and providing hands-on assistance. Data suggests that support tailored to a specific condition improves health outcomes compared to more generic support. Thus, it may be postulated that when it comes to managing chronic diseases, assistance that is tailored to the individual condition or treatment regimen may have a more pronounced effect on self-management behavior compared to more generic forms of support [69]. Coping with visual impairment and striving to preserve autonomy in everyday tasks can be an extremely difficult experience. The capacity to cultivate novel personal resources to offset the impairment resulting from visual loss is predominantly contingent upon the efficacy of psychological adaptation. Psychological adaptation to vision loss refers to the cognitive and behavioral process by which an individual effectively adjusts to the challenges and constraints brought about by the loss of vision [19]. Individuals are highly susceptible to emotional distress and social isolation throughout the adaptation process; consequently, they may develop psychological issues including depression, anxiety, and sleep disorders. Frequently, these patients’ psychological issues serve as an additional burden of disability, impeding their ability to be active and reintegrate into society [71, 72]. The perception of social support is prominent among the determinants correlated with enhanced adaptation [19]. Interventions designed to increase the support of the patient’s friends and family, in addition to the establishment of community peer support groups, can also benefit from social support. Social support from family and acquaintances had a substantial effect on psychological well-being and adaptation to vision loss [24]. Social support can indirectly influence self-management by enhancing self-efficacy. Additional consequences may potentially arise through alternative psychological mechanisms. Being part of a supportive social network can have positive impacts on motivation, coping mechanisms, and psychological well-being. Highly motivated individuals, who have strong morale, or experience less depression may participate in situations linked to their illness [73–75] and exhibit more activation.

As noted in Social Cognitive Theory, the engagement of individuals with chronic illnesses in the self-management process occurs within a context that includes formal healthcare providers, informal social network members, and the physical environment (e.g., housing, air quality). All of these contextual factors have the potential to significantly influence self-management behavior, either directly or indirectly through self-efficacy [74]. The Personalized Patient Activation and Empowerment Model (P-PAE) is a comprehensive concept that encompasses several aspects such as social and physical settings, patients, healthcare professionals, communities, and the broader healthcare delivery system. The model prioritizes patients as the focal point of the system and employs patient-centered outcome research theory to elucidate how individualized patient activation and empowerment may be achieved [76]. Furthermore, the environment encompasses a wide range of elements that might either impede or facilitate patients’ engagement, performance, and entitlement to preserve their dignity [77]. For example, the International Classification of Functioning, Disability, and Health (ICF) defines environmental factors as “those that constitute the physical, social, and behavioral environment in which people live and lead their lives.” The impact of the environment on an individual’s life and ability to function depends on the degree of support or demand (e.g., accessibility, usability) that the physical environment may have [78]. For example, person-environment adaptation theories explain that the adequacy of the fit between a person’s functional abilities and their environment can affect a person’s level of independence, participation, and overall health and well-being [77]. In our research, it was seen that patient activation levels of clients with vision loss were related to the environment sub-dimension of quality of life and that the environment was an effective factor in the established regression model. The ICF, which is also accepted by the World Health Organization (WHO), states that environmental factors are very important for patient health outcomes. Therefore, health professionals should integrate environmental factors into their assessments and goal-setting to encourage patient participation [78]. Moreover, the current trend towards short-term hospital stays and ongoing rehabilitation and care at home for people with complex health problems requires greater involvement of the environment in health-related communication throughout the care process. Small changes in the physical environment can have large effects on behavior and can be used in environmental, self-management, and chronic disease research [79].

Patient activation can be considered as the operationalisation of the concept of patient empowerment or patient self-efficacy in the focus of chronic condition management in recent years. It also provides additional benefits in terms of more effective self-care and tailoring services, as well as greater efficiency [80]. Globally, there is a growing awareness that patients need to become more active and effective managers of their health and health care in terms of strategies to improve health care quality [58]. The increasing prevalence and duration of visual impairment cause an increased burden of self-management and the need for more support for visually impaired individuals and their families [81]. For this reason, activities related to patient activation are extremely important for visually impaired individuals. It is critical for visually impaired individuals to have access to education and supportive interventions in order to increase their skills and confidence in the management of their own health problems in order to gain awareness of healthy living, self-management and self-efficacy, and to enable their families to acquire behaviours for health promotion [82]. Programmes to be planned to ensure the development of patient activation in visually impaired individuals will also address medical management (disease information, drug management, etc.), role management (management of daily family and work-related functions) and emotion management (stress management, problem solving and adaptation skills, etc.) [81, 83, 84]. In addition, it is very important to encourage social support, such as peer support, inter-patient support and coaching support, that contributes to improved self-management behaviour [19, 85]. Finally, these programmes to improve patient activation should focus on the priorities of the visually impaired client; that is, they should be presented individually and prioritise concepts like self-management, self-efficacy, healthy living awareness, supportive social relations and environment in light of the findings of our study.

### Strengths and limitations

Our study is the first study in which variables, including self-management, self-efficacy, health awareness, quality of life related to social relationships and the environment in visually impaired individuals, are included and comprehensively examined. The study was conducted in a secondary health care centre where outpatient or inpatient diagnosis, treatment and rehabilitation services are provided. Individuals with vision loss from many provinces of Turkey apply, which increases the generalisability of our study results. However, the findings of the individuals who have applied to health services may not fully reflect the characteristics of the visually impaired population, which includes individuals who are not health care recipients and may be biased in favour of participants who have applied for health services in terms of activation level. The use of self-reported questionnaires for assessments may result in misclassification due to socially desirable responses. Although our study shows the relationship between the investigated variables and activation, it includes a cross-sectional design that does not allow the evaluation of temporal effects or causality potential. In order to better understand the effects of factors affecting patient activation over time, longitudinal studies are needed. Even though PAM is extensively used for chronic health conditions, it is limited to the perceived self-assessment of the patient’s ability to manage self-care, rather than the direct measurement of self-management behaviour itself [86]. In addition, patient activation may require situation-specific knowledge and skills in situations related to vision loss. Even though PAM has been used in a study by Morse and Seiple [16] to measure activation in individuals with visual impairment (item reliability was 0.88, and person reliability was 0.86), it can be discussed in further research whether it is an appropriate measure for visually impaired individuals.

## Conclusion

This study showed that visually impaired individuals are activated at the level of taking action according to PAM, and it showed that marital status, self-management, self-efficacy, health awareness, quality of life related to social relationships and the environment greatly affect patient activation. These results reflect the importance of addressing self-management, self-efficacy, health awareness, quality of life related to social relationships and the environment in achieving better health outcomes in visually impaired individuals. However, although including patient activation within the scope of providing healthcare services to visually impaired individuals has the potential to target quality of life with the existing chronic condition by improving the health behaviours of individuals, additional evidence is needed to better understand the role of patient activation in visually impaired individuals.

## Data Availability

The datasets used and/or analysed during the current study are available from the corresponding author on reasonable request.

## References

[1] Klein R, Klein BE (2013). The prevalence of age-related eye diseases and visual impairment in aging: current estimates. Invest Ophthalmol Vis Sci.

[2] WHO. Visual impairment and blindness. https://www.who.int/news-room/fact-sheets/detail/blindness-and-visual-impairment. 2013.

[3] Christ SL, Zheng DD, Swenor BK, Lam BL, West SK, Tannenbaum SL (2014). Longitudinal relationships among visual acuity, daily functional status, and mortality: the Salisbury Eye evaluation study. JAMA Ophthalmol.

[4] Crews JE, Chou CF, Zhang X, Zack MM, Saaddine JB (2014). Health-related quality of life among people aged ≥ 65 years with self-reported visual impairment: findings from the 2006–2010 behavioral risk factor surveillance system. Ophthalmic Epidemiol.

[5] Demir FE, Ozdemir S (2016). A comparison of social skills of students with visual impairments and typically developing students. Int E-J Adv Educ.

[6] Luszczynska A, Gutiérrez-Doña B, Schwarzer R (2005). General self‐efficacy in various domains of human functioning: evidence from five countries. Int J Psychol.

[7] O’Conor R, Smith SG, Curtis LM (2018). Mild visual impairment and its impact on self-care among older adults. J Aging Health.

[8] Owsley C (2016). Vision and Aging. Annu Rev Vis Sci.

[9] Roh M, Selivanova A, Shin HJ (2018). Visual acuity and contrast sensitivity are two important factors affecting vision-related quality of life in advanced age-related macular degeneration. PLoS ONE.

[10] Greene J, Hibbard JH, Sacks R, Overton V, Parrotta CD (2015). When patient activation levels change, health outcomes and costs change, too. Health Aff.

[11] Hibbard JH, Mahoney ER, Stockard J, Tusler M (2005). Development and testing of a short form of the patient activation measure. Health Serv Res.

[12] Deen D, Lu WH, Rothstein D, Santana L, Gold MR (2011). Asking questions: the effect of a brief intervention in community health centers on patient activation. Patient Educ Couns.

[13] Hibbard JH, Stockard J, Mahoney ER, Tusler M (2004). Development of the patient activation measure (PAM): conceptualizing and measuring activation in patients and consumers. Health Serv Res.

[14] Hibbard JH, Greene J, Tusler M (2009). Improving the outcomes of disease management by tailoring care to the patient’s level of activation. Am J Manag Care.

[15] Grembowski D, Patrick D, Diehr P, Durham M, Beresford S, Kay E, Hecht (1993). J self-efficacy and health behavior among older adults. J Health Soc Behav.

[16] Morse AR, Seiple W (2021). Activation in individuals with vision loss. J Health Psychol.

[17] Evers AW, Kraaimaat FW, van Lankveld W, Jongen PJ, Jacobs JW, Bijlsma JW (2001). Beyond unfavorable thinking: the illness cognition questionnaire for chronic diseases. J Consult Clin Psychol.

[18] Nyman SR, Dibb B, Victor CR, Gosney MA (2012). Emotional well-being and adjustment to vision loss in later life: a meta-synthesis of qualitative studies. Disabil Rehabil.

[19] Senra H, Barbosa F, Ferreira P, Vieira CR, Perrin PB, Rogers H (2015). Psychologic adjustment to irreversible vision loss in adults: a systematic review. Ophthalmology.

[20] Verhoof EJ, Maurice-Stam H, Heymans HS, Evers AW, Grootenhuis MA (2014). Psychosocial well-being in young adults with chronic illness since childhood: the role of illness cognitions. Child Adolesc Psychiatry Ment Health.

[21] Heijmans M, de Ridder D (1998). Assessing illness representations of chronic illness: explorations of their disease-specific nature. J Behav Med.

[22] Owsley C, McGwin G, Scilley K, Dreer LE, Bray CR, Mason JO (2006). Focus groups with persons who have age-related macular degeneration: emotional issues. Rehabil Psychol.

[23] Nyman SR, Margot AG, Christina RV. Psychosocial impact of visual impairment in working-age adults. Br J Ophthalmol (2010). 10.1136/bjo.2009.164814 19850584

[24] Reinhardt JP (2001). Effects of positive and negative support received and provided on adaptation to chronic visual impairment. Appl Dev Sci.

[25] Sabel BA, Wang J, Cárdenas-Morales L, Faiq M, Heim C (2018). Mental stress as consequence and cause of vision loss: the dawn of psychosomatic ophthalmology for preventive and personalized medicine. EPMA J.

[26] Bodenheimer T, Lorig K, Holman H, Grumbach K (2002). Patient self-management of chronic disease in primary care. JAMA.

[27] Holman H, Lorig K (2004). Patient self-management: a key to effectiveness and efficiency in care of chronic disease. Public Health Rep.

[28] Street RL, Gordon HS, Ward MM, Krupat E, Kravitz R (2005). L.Patient participation in medical consultations: why some patients are more involved than others. Med Care.

[28] Jiang S, Hong YA (2018). Mobile-based patient-provider communication in cancer survivors: the roles of health literacy and patient activation. Psychooncology.

[30] Kato A, Fujimaki Y, Fujimori S, Isogawa A, Onishi Y, Suzuki R (2020). How self-stigma affects patient activation in persons with type 2 diabetes: a cross-sectional study. BMJ Open.

[31] McCabe PJ, Stuart-Mullen LG, McLeod CJ, Byrne T, Schmidt MM, Branda ME, Griffin JM (2018). Patient activation for self-management is associated with health status in patients with atrial fibrillation. Patient Prefer Adherence.

[32] McCann RM, Jackson AJ, Stevenson M, Dempster M, McElnay JC, Cupples ME (2012). Help needed in medication self-management for people with visual impairment: case-control study. Br J Gen Pract.

[33] Kosar C, Besen DB (2019). Adaptation of a patient activation measure (PAM) into Turkish: reliability and validity test. Afr Health Sci.

[34] Mezo P (2008). The Self-Control and Self-Management Scale (SCMS): development of an adaptive Self-Regulatory Coping skills Instrument. J Psychopathol Behav Assess.

[35] Ercoşkun M (2016). Özkontrol-Özyönetim Ölçeğinin (ÖKYÖ) Türk Kültürüne Uyarlanması: Geçerlik ve Güvenirlik Çalışması (Adaptation of Self-Control and Self-Management Scale (SCMS) into Turkish culture: a study on reliability and validity). Educational Sciences: Theory Pract.

[36] Mezo P, Short M (2012). Construct validity and Confirmatory Factor Analysis of the self-control and self-management scale. Can J Behav Sci.

[37] Xue G, Sun X (2011). Construction and validation of self-management scale for undergraduate students. Creative Educ.

[38] Yildirim F, Ozgur Ilhan I (2010). The validity and reliability of the General Self-Efficacy Scale-turkish form. Türk Psikiyatri Dergisi.

[39] Özer E, Yılmaz N (2020). Healthy life awareness: a Scale Development Study. J Traditional Med Complement Ther.

[40] World Health Organization [WHO] (1998). Development of the World Health Organization WHOQOL-BREF quality of life assessment. Psychol Med.

[41] Eser E, Fidaner H, Fidaner C, Eser SY, Elbi H, Göker E. (1999). WHOQOL-100 ve WHOQOL-BREF’in psikometrik özellikleri. Psikiyatri Psikoloji Psikofarmakoloji (3P) Dergisi, 7(Suppl 2), 23–40.

[42] Eser SY, Fidaner H, Fidaner C, Elbi H, Eser E, Göker E. (1999). Measure of quality of life WHOQOL-100 and WHOQOL-Bref. 3P Dergisi, 7(2 Suppl), 5–13.

[43] Alpar R (2011). Uygulamalı Çok Değişkenli İstatistiksel Yöntemler: Applied Multivariate Statistical methods.

[44] Field AP. Discovering Statistics Using IBM SPSS Statistics: And Sex and Drugs and Rock ‘n’ Roll, 4th edition. Los Angeles: SAGE Publications; 2013.

[45] Hoerl AE, Kennard RW (1970). Ridge Regression: biased estimation for nonorthogonal problems. Technometrics.

[46] Montgomery DC, Peck EA, Vining GG. Introduction to Linear Regression Analysis, 3rd Edition, John Wiley & Sons, New York, 2001.

[47] Vinod HD, Ullah A (1981). Recent advances in regression methods.

[48] Hoerl AE, Kennard RW (1970). Ridge regression: applications to nonorthogonal problems. Technometrics.

[49] Khalaf G, Iguernane M (2016). Multicollinearity and a ridge parameter estimation approach. J Mod Appl Stat Methods.

[50] Marquardt D, Snee R (1975). Ridge Regression in Practice. Am Stat.

[51] Hibbard JH, Mahoney ER, Stock R, Tusler M (2007). Do increases in patient activation result in improved self-management behaviors?. Health Serv Res.

[52] Hendriks M, Rademakers J (2014). Relationships between patient activation, disease-specific knowledge and health outcomes among people with diabetes; a survey study. BMC Health Serv Res.

[53] Hendriks SH, Hartog LC, Groenier KH, Maas AH, van Hateren KJ, Kleefstra N, Bilo HJ (2016). Patient activation in type 2 diabetes: does it differ between men and women?. J Diabetes Res.

[54] Rademakers J, Nijman J, van der Hoek L, Heijmans M, Rijken M (2012). Measuring patient activation in the Netherlands: translation and validation of the American short form patient activation measure (PAM13). BMC Public Health.

[55] Wilkinson TJ, Memory K, Lightfoot CJ, Palmer J, Smith AC (2021). Determinants of patient activation and its association with cardiovascular disease risk in chronic kidney disease: a cross-sectional study. Health Expect.

[56] Mayberry R, Willock RJ, Boone L, Lopez P, Qin H, Nicewander D (2010). A high level of patient activation is observed but unrelated to Glycemic Control among adults with type 2 diabetes. Diabetes Spectr.

[57] Ryvicker M, Feldman PH, Chiu YL, Gerber LM (2013). The role of patient activation in improving blood pressure outcomes in black patients receiving home care. Med Care Res Rev.

[58] Greene J, Hibbard JH (2012). Why does patient activation matter? An examination of the relationships between patient activation and health-related outcomes. J Gen Intern Med.

[59] Bos-Touwen I, Schuurmans M, Monninkhof EM, Korpershoek Y, Spruit-Bentvelzen L, Ertugrul-van der Graaf I (2015). Patient and disease characteristics associated with activation for self-management in patients with diabetes, chronic obstructive pulmonary disease, chronic heart failure and chronic renal disease: a cross-sectional survey study. PLoS ONE.

[60] Newland P, Lorenz R, Oliver BJ (2021). Patient activation in adults with chronic conditions: a systematic review. J Health Psychol.

[61] DiMatteo MR (2004). Social support and patient adherence to medical treatment: a meta-analysis. Health Psychol.

[62] Qadir F, Khalid A, Haqqani S, Medhin G (2013). The association of marital relationship and perceived social support with mental health of women in Pakistan. BMC Public Health.

[63] Do V, Young L, Barnason S, Tran H (2015). Relationships between activation level, knowledge, self-efficacy, and self-management behavior in heart failure patients discharged from rural hospitals. F1000Res.

[64] Bourbeau J, Nault D, Dang-Tan T (2004). Self-management and behaviour modification in COPD. Patient Educ Couns.

[65] Anekwe TD, Rahkovsky I, Self-Management (2018). A Comprehensive Approach to Management of Chronic conditions. Am J Public Health.

[66] Diotaiuti P, Petruccelli F, Rea L, Zona AM, Verrastro V (2014). The role of self-control on Mood States and Health anxiety in a sample of Blind and visually impaired people. Psychology.

[67] Rodriguez HP, Poon BY, Wang E, Shortell SM (2019). Linking practice adoption of Patient Engagement strategies and relational coordination to patient-reported outcomes in Accountable Care Organizations. Milbank Q.

[68] Ozbay F, Johnson DC, Dimoulas E, Morgan Iii CA, Charney D, Southwick S (2007). Social support and resilience to stress: from neurobiology to clinical practice. Psychiatry (Edgmont).

[69] Gallant MP (2003). The influence of social support on chronic illness self-management: a review and directions for research. Health Educ Behav.

[70] Sztonyk B, Formella Z (2020). The role of social support in contributing to posttraumatic growth in persons with vision impairment. Health Psychol Rep.

[71] Verstraten PFJ, Brinkmann WLJH, Stevens N, Schouten JSAG (2005). Loneliness, adaptation to vision impairment, social support and depression among visually impaired elderly. Int Congress Ser.

[72] Kempen GI, Ballemans J, Ranchor AV, van Rens GH, Zijlstra GR (2012). The impact of low vision on activities of daily living, symptoms of depression, feelings of anxiety and social support in community-living older adults seeking vision rehabilitation services. Qual Life Res.

[73] Dwarswaard J, Bakker EJ, van Staa A, Boeije HR (2016). Self-management support from the perspective of patients with a chronic condition: a thematic synthesis of qualitative studies. Health Expect.

[74] Schulman-Green D, Jaser SS, Park C, Whittemore R (2016). A metasynthesis of factors affecting self‐management of chronic illness. J Adv Nurs.

[75] Toyoshima A, Martin P, Sato S, Poon LW (2018). The relationship between vision impairment and well-being among centenarians: findings from the Georgia Centenarian Study. Int J Geriatr Psychiatry.

[76] Chen J, Mullins CD, Novak P, Thomas SB (2016). Personalized strategies to activate and empower patients in Health Care and Reduce Health disparities. Health education & behavior: the official publication of the Society for. Public Health Educ.

[77] Kylén M, Schön UK, Pessah-Rasmussen H, Elf M. Patient participation and the environment: A scoping review of instruments. International Journal of Environmental Research and Public Health. 2022; 19(4): 2003. 10.3390/ijerph19042003 PMC8872044 35206191

[78] World Health Organization (2001). International Classification of Functioning, Disability and Health; World Health Organization.

[79] Adams RJ. Improving health outcomes with better patient understanding and education. Risk Manage Healthc Policy. 2010; 61–72. 10.2147/RMHP.S7500 PMC3270921 22312219

[80] Segal LA (2021). Big WIN in Texas: demonstrating the benefits of patient self-efficacy and patient activation. Value Health.

[81] Rees G, Xie J, Chiang PP, Larizza MF, Marella M, Hassell JB, ... & Lamoureux ELA. Randomised controlled trial of a self-management programme for low vision implemented in low vision rehabilitation services. Patient Educ Couns. 2015;98(2):174-181. 10.1016/j.pec.2014.11.008 25481576

[82] Ghasemi A, Karimi Moonaghi H, Mohajer S, Mazlom SR, Shoeibi N (2018). Effect of self-management educational program on vision-related quality of life among elderly with visual impairment. Evid Based Care J.

[83] Zhi-Han L, Hui-Yin Y, Makmor-Bakry M (2017). Medication Handling challenges among visually impaired Population. Arch Pharm Pract.

[84] Rees G, Ponczek E, Hassell J, Keeffe JE, Lamoureux EL (2010). Psychological outcomes following interventions for people with low vision: a systematic review. Expert Rev Ophthalmol.

[85] Holloway EE, Xie J, Sturrock BA, Lamoureux EL, Rees G (2015). Do problem-solving interventions improve psychosocial outcomes in vision impaired adults: a systematic review and meta-analysis. Patient Educ Couns.

[86] Goodworth MC, Stepleman L, Hibbard J, Johns L, Wright D, Hughes MD, Williams MJ (2016). Variables associated with patient activation in persons with multiple sclerosis. J Health Psychol.

